# Creating security for the child in grief: a delicate task for the school nurse – findings from a web-based survey on school nurses’ experiences

**DOI:** 10.1177/17449871251401025

**Published:** 2026-01-09

**Authors:** Nadja El Gharbawi, Anna Karlsson, Eva Clausson, Katarina Sjövall

**Affiliations:** District Nurse, Health Care Center Kristianstad, Kristianstad, Sweden; District Nurse, Health Care Center Vellinge, Vellinge, Sweden; Senior Lecturer, Faculty of Health Sciences, Kristianstad University, Kristianstad, Sweden; Senior Lecturer, Faculty of Health Sciences, Kristianstad University, Kristianstad, Sweden

**Keywords:** bereavement, child, childhood grief, grief, school nursing, school support

## Abstract

**Background:**

The school nurse is tasked with promoting health for children. However, the school nurse’s role in meeting the needs of children experiencing grief following the death of a family member remains unexplored.

**Aim:**

The aim of this study was to investigate the experiences of school nurses in supporting children in grief following the death of a family member.

**Methods:**

The study was conducted as a cross-sectional study employing qualitative and quantitative approaches through a web-based questionnaire (*n* = 62 school nurses).

**Results:**

A majority (90%) of the school nurses felt secure in their professional role, most of them (93%) had experience of meeting children with grief. Qualitative findings revealed an overarching theme *Creating security for the child* including two themes: *Security for the school nurse* which included two subthemes- *Feeling secure in the meeting* and *Feeling security in the team; and Security for the child* which included two subthemes *– Creating a secure environment for the child* and *Being there for the child*.

**Conclusion:**

Our findings indicate that school nurses can play an important role for the child in creating a secure place for the grieving child in school. Further research should focus on school nurses’ more specific needs for preparational training and support.

The school nurse has good knowledge of supporting students in difficult situations, but needs and life situation need to be taken into account (Golsäter et al., 2017). The death of a family member, such as a parent or sibling, can be the most difficult and dramatic event in a child’s life and entails daily challenges in connection with the loss (Dyregrov et al., 2020). Social distancing and difficulties in maintaining relationships with peers are often triggered by the feeling of alienation after a loss (Dyregrov et al., 2020). Similarly, grief can negatively affect school performance for some time after the loss and may lead to reduced opportunities for future academic studies.

Denial, anger, bargaining, depression and acceptance were described in the 60th by Kübler-Ross (1997) as a model for understanding different phases of grieving. The model is applicable for describing children’s grief as well as for adults. However, it is emphasised that there are no fixed phases for children to follow through their grief, and their grief is highly individual (Dyregrov et al., 2020; Farella Guzzo and Gobbi, 2023; Kübler-Ross 1997). Children’s grief can be expressed in different ways. Common grief reactions are fear, sadness, anger, bodily pain, anxiety, guilt, and shame. From preschool age, fantasy and reality can be difficult to distinguish and the understanding of death is limited (D'Alton et al., 2022). Excessive helpfulness towards adults can be an expression of hope for a normalisation of existence. Another common response among younger children in grief is anger and aggressive behaviour (D'Alton et al., 2022).

From the age of 10, children have well-developed rational and logical thinking (Dyregrov and Lytje, 2022) and seek approval and mastery (Erikson, 1977). Unlike the younger child, death is a concept that is possible to understand and implies an eternal and irreversible condition. For children who have lost a family member, the awareness of their own mortality or the fear of losing their guardians can therefore give rise to strong reactions (D'Alton et al., 2022).

Adolescence is a time characterised by liberation and exploration of, as well as, forming their identity (Erikson, 1977). In the case of the loss of a family member, it can mean an interruption in the natural process of identity search (Dyregrov and Lytje, 2022; D’Alton et al., 2022). For example in D’Alton et al.’s (2022) study, existential questions about personal worth and loss of meaning were expressed in teenagers who had lost a sibling. Furthermore, compared to younger children, teenagers had a higher rate of somatic or physical complaints 1 year after the death of a family member (D’Alton et al., 2022). A recent study by Bylund-Grenklo et al. (2021) found that a majority of young children felt that they could not channel their grief properly during the acute phase of grieving. Instead, they numbed their feelings or held back their grief to spare family members. A literature review showed that teenagers who lost a family member might develop mood disorders, have increased suicidal ideations, anxiety, insomnia, and increased risk behaviours and use of drugs and alcohol (Farella Guzzo and Gobbi, 2023).

A prolonged grief reaction in children and young people can be associated with ill health. In the long run, it can create obstacles to returning to normal functioning (Melhem et al., 2011). The death of a family member, regardless of whether unexpected or anticipated, might lead to a negative impact on the mental health of children and young people (Farella Guzzo and Gobbi, 2023). Concentration difficulties and low motivation to study are not unusual consequences of a loss, and these can limit the possibility of continued development and promotion of health (Liu et al., 2022). Children in grief are better able to face difficulties and upheaval events in stable and safe environments (Ellis et al., 2013). As school is a major part of children’s lives, it plays a vital role in recognising their individual needs and providing appropriate bereavement support (Auman, 2007). This highlights the important role of student health in contributing to making school a safe place for the grieving child with a permissive climate and acceptance of the child’s needs.

In Sweden, a student health team conducts medical, psychological, psychosocial, and special educational interventions and consists of a school doctor, a school nurse, a psychologist, a counsellor, and a special education teacher. The student health team focuses on health promotion and prevention of illness, and also on supporting the students’ development towards the goals of education (National Board of Health and Welfare, 2023). The school nurse’s cross-professional collaboration within the student health team, with educators, and with the family is central to support children and their families in difficult situations (Golsäter et al., 2017).

The school nurse is tasked with promoting health and creating health-promoting environments for the students at school. Practising as a school nurse requires in-depth knowledge of nursing and public health science (National Association of School Nurses and Swedish Nurses’ Association, 2025). The scientific approach must underpin the nursing work in the school nurse’s work, which in practice means supporting students to independently preserve and promote health. From a public health perspective, the school nurse works both systematically and in a goal-oriented manner to achieve equal health among students (National Association of School Nurses and Swedish Nurses’ Association, 2025). The school nurse’s specialist knowledge in nursing is necessary for identifying ill health and preventing disease. In Kostenius’ (2023) interview study with school nurses, the routine health talks that the school nurse undertakes were described as a success factor for meeting each student’s needs. The experience that the students’ threshold was lower when it came to talking about difficult things like bullying and exclusion was clear during the health talks. Golsäter et al. (2017) problematised the school nurse’s exclusion when it comes to the transfer of information regarding students whose guardians have suffered a terminal illness leading to death. Not having information on expected death in the family reduced the school nurse’s ability to show her support for the student.

The school nurse plays an important role in promoting health and preventing ill health when meeting children in grief. Nursing research is limited in the area, therefore it is important to study the subject and reduce the knowledge gap. The study can increase understanding of the school nurse’s work, thereby clarifying the profession’s role in supporting children in grief.

## Purpose

The purpose of this study was to investigate the experiences of school nurses in supporting children in grief following the death of a family member.

## Method

This study was undertaken as a cross-sectional study using a web-based survey with multiple choice questions and open questions. Answers were analysed quantitatively and qualitatively. This study is reported in accordance with the Consolidated Criteria for Reporting Qualitative Research (COREQ) guidelines (Tong et al., 2007).

### Participants and participant recruitment

School nurses working in public or independent schools in Sweden, active within a student health team serving students aged 6–19 years, were eligible for participation. School nurses at schools within urban areas, the countryside, and cities were approached to participate. Participants were recruited via a Facebook forum for school nurses only, via mail sent to school nurses in several municipalities in Sweden, via mail sent to principals at schools in southern Sweden, and at a conference ‘*Health and Well-being of Children and Young People*’ (Kristianstad University, October 2023). The study was presented with a written information letter, and the web-based survey was accessed by a QR code (a two dimensional Quick Response code). Participants were informed before starting that continuing with the survey was interpreted as consent to participate.

### Data collection

The web-based survey was constructed with 17 questions, based on two previous national surveys in which school nurses assessed the health of school children (Clausson et al., 2008, Ellertsson et al., 2017). The opening questions concerned demographic data about participants, and the rest of the questions were open questions about participants’ experiences of supporting school children in grief after the death of a close family member. A pilot study was performed to test the web-based survey. Three formerly active school nurses, now working in universities, were invited and agreed to participate. They were asked to give their view on the information text and on the questions. After feedback from the three, the introduction text was revised regarding the estimated length of time for answering the survey. Data collection continued until a variation was reached regarding participants’ characteristics (length of experience in school nursing) and also a variation in data (a variation in how participants experienced meeting children in grief). When variations in data and participants’ characteristics were achieved, information power was assessed as sufficient, giving a sample size of 62 participants. The web-based survey was open from September 2023 to January 2024.

### Data analysis

Descriptive statistics were used to describe demographical data. The written answers to the open questions were analysed with content analysis according Graneheim and Lundman (2004). In the first step of the analysis, all answers were read individually by the first two authors (N.E.G, A.K.) several times to get a sense of the content. In the second step, the first two authors worked together to identify and compile content on a manifest level. The rest of the text was compiled and analysed as a whole. In the third step, meaning units were identified, representing condensed parts of the text with the essence of the meaning. In the fourth step, the meaning units were labelled with codes that described what they were about. Codes were compared for similarities and differences. Based on codes with similar meanings and content, themes were identified from the latent content. To ensure credibility, there was an ongoing discussion between all the authors throughout the whole process of analysis, related to the meaning units and the codes, with mutual reflections upon emerging subthemes and themes. The subthemes and themes were finalised once a consensus was reached among all authors. Finally, one overarching theme was identified that reflected a common thread in the content of all themes. Trustworthiness was sought through the whole process of work with the study by considering credibility, dependability, confirmability and transferability (Shenton, 2004). To strengthen the dependability of the study, the questions in the web-based survey were tested by three former school nurses now working in research and education. After feedback from them, a minor revision of the information text in the survey was made. To strengthen credibility in the analysis process, all the authors had an ongoing discussion reflecting upon emerging subthemes and themes. The four authors have different backgrounds and experiences in nursing, meeting grieving people and in school nursing, contributing to ensuring the confirmability of the study. The first two authors (N.E.G and A.K.) are experienced nurses with training for school nursing. The third author (E.C.) is an experienced school nurse working with research and teaching in school nursing. The fourth author (K.S.) is an oncology nurse with many years’ experiences in palliative care and research. To strengthen confirmability further, quotes were used to illustrate school nurses’ experiences. Finally, transferability was considered in striving for participants with a broad variation in age, their experiences of working with children in grief, their area of work (smaller or larger school), and the age of the children with whom they work.

#### Ethical considerations

This study was approved by The Health Sciences Ethics Council at Kristianstad University before the recruitment of participants (dnr U2023-2.1.12-763). Written information about the study, its purpose and design, was provided to each participant at the start of the web survey. Continuing the web survey was interpreted as informed consent to participate. All collected data were anonymous.

## Results

The results are based on answers from 62 school nurses who agreed to participate in the study. A majority were trained as district nurses or paediatric nurses. Most of the participants were women, operating in a public school in a city or smaller urban area. The participants’ demographic data is presented in Table 1.

**Table 1.** Demographic data participants.

Demographic variables	*n* (%)
Age	
25–34 years	2 (3)
35–44 years	18 (9)
45–54 years	24 (39)
55–64 years	16 (26)
65 years	2 (3)
Gender	
Male	1 (2)
Female	61 (98)
Specialist nurse education	
District nurse	30 (48)
Paediatric nurse	24 (39)
School nurse	6 (10)
Other	2 (3)
Experience as school nurse	
Mean (variants)	9 years (2 months–32 years)
Number of children	
Mean (variants)	400 (65–600)
Age of children	*n* (%)
6–12 years	26 (42)
6–15 years	23 (37)
16–19 years	5 (8)
Mixed	1 (2)

Most of the school nurses (93%) had previous experience of meeting and supporting children experiencing grief, and children of different ages who had lost a family member due to illness or suicide. Most of them (90%) felt secure in their professional role, and they had previous experience of meeting children in grief due to the loss of a family member. A preparedness for how to meet with children in grief had been acquired through nursing and specialist nursing education. Some were offered internal training by the employer about crisis reactions, trauma-aware care, and treatment of families’ grief. However, some school nurses experienced a lack of knowledge about child bereavement at different ages. Most of the school nurses (95%) felt that they had well-functioning cooperation within the student health team about children in grief.

### Creating security for the child in grief

**Figure 1.**
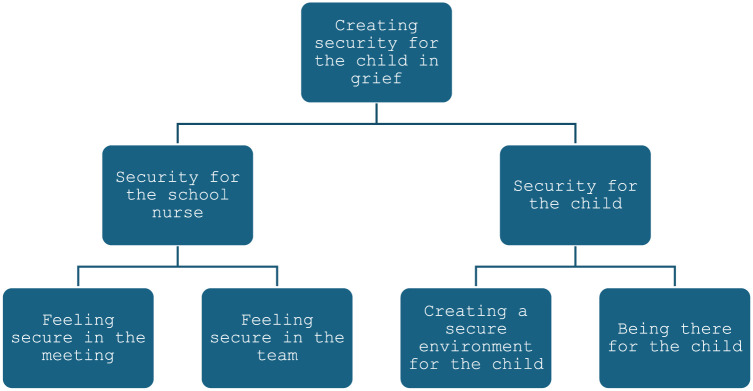
School nurses’ experiences of meeting a child in grief after the loss of a family member.

School nurses experienced that creating security was a central part of their role whenever there was a child in grief due to the loss of a family member. It was based on feeling secure in the meeting with the child, a feeling that related to the school nurse’s previous training and experiences of grief. It also included the feeling of security in the team. Furthermore, school nurses stressed the importance of helping school remain a secure environment for the child, with as much normality as possible. The role of the school nurse was experienced as being an accessible adult for the bereaved child in school. The overarching theme included two themes. The first theme *Security for the school nurse* included two subthemes, *Feeling* s*ecure in the meeting* and *Feeling security in the team.* The second theme *Security for the child* included two subthemes *Creating a secure environment for the child* and *Being there for the child*. The overarching theme, themes and included subthemes are presented in Figure 1.

#### Security for the school nurse

##### Feeling secure in the meeting

Security in the meeting was based on a feeling of being confident in oneself and in the profession of being a school nurse. Becoming confident was related to personally experienced grief and previous experience from meeting with bereaved children, both privately and professionally. Knowledge about supporting bereaved individuals had been acquired from nursing education and prior clinical experience. All previous experience of caring for terminally ill and dying people, whether it was from adult or paediatric care, contributed to having a ‘toolbox’ when meeting bereaved children:

I feel quite safe in this as I worked for 10 years in an emergency department where it was common that I had to take care of people (both children and adults) who had lost someone close. (Participant no. 8, female, 10 years of professional experience, school nurse)

Feeling comfortable with questions dealing with death and grief was developed during work as a school nurse. Initially, it was challenging, but the interaction became easier as more experience was gathered over time:

Since I have long experience, I feel safe in meeting children in grief. At the beginning of my career as a school nurse, I thought it was difficult, but because I myself am comfortable with questions about illness, death and grief, it makes it easier when meeting children in grief. (Participant no. 7, female, 17 years of professional experience)

However, even if feeling confident and comfortable as a professional, the meeting with the child could be both difficult and challenging. As it did not happen that often, it was experienced as difficult to become really secure in the situation. Some school nurses said that even though they had long experience and were not afraid of meeting the child with grief, they never got used to it.

Because it happens so rarely, it is hard to feel secure

(Participant no. 31, female, 5 years of professional experience)

##### Feeling secure in the team

School nurses’ meetings with children in grief were experienced to be emotionally engaging. They stressed the importance of having a secure workplace with close colleagues to discuss cases with and to rely on:

. . .Sometimes you become very emotionally involved, sometimes less so. It is important that you have a safe place to work and that you can discuss cases with close colleagues. (Participant no. 14, female, 10 years of professional experience)

Having colleagues of other professions in the team to talk to contributed to feeling secure in one’s own profession as a school nurse. Peer support in work with bereaved children was important and could be provided by teachers, school curators, school psychologists, school leaders, and doctors.

I have a really well-functioning student health team and feel supported by the different professionals (including two school nurses, colleagues, and a school doctor) (Participant no. 13, 5 years of professional experience)

Teamwork was especially used in the work of mapping the situation and the needs of the bereaved child. Sometimes external resources such as social workers and priests were involved. However, when teamwork was not working as expected it led to feelings of uncertainty and insecurity. Differences in the view of what support to offer and by whom could result in a lack of effective teamwork.

I wish our school counsellor took a bigger role in this, but sometimes she hints that it’s not ‘school related’ and therefore not really her responsibility . . .

(Participant no. 17, woman, seven years of professional experience)

#### Security for the child

##### Creating a secure environment for the child

School nurses experienced that the bereaved child needed the school to represent normality and provide a secure environment. The school nurse reception area should provide a secure and enabling environment where it was possible to grieve and find comfort in silence but should also serve as a free zone to take a break from grieving, away from the rest of the school. Representing normality meant helping the child preserve the feeling of being part of the crowd and not standing out. Allowing the child to be part of the crowd when the child wanted was a way for the school nurse to create security:

For some, I am the one who constitutes the ‘normal’ where the student, just like everyone else, gets vaccines, and takes examinations. Then I offer security in that I let the student be part of the crowd when they wish to. . . (Participant no. 47, woman, five years of professional experience)

Returning to school was an opportunity for the child to reunite with friends, which was another way of getting back to normality. However, sometimes the child needed support in their contact with friends when returning to school. The school nurse could assist in informing the class and could also guide friends in the schoolyard when they became over caring.

Ask the student/family what they want from school, sometimes they just want “everything to be as usual” at school

(Participant no. 20, female, 9 years of experience)

##### Being there for the child

Being there for the child was about being accessible and humble and respecting the wishes of the child.

The role of the school nurse was experienced as being an accessible adult for the bereaved child in the moment as well as over time. It included accessibility by being present in the classroom and schoolyard, having an open reception area and having scheduled meetings or sending regular texts (SMS, Short Message Service). Apart from being physically present it was also about having the courage to talk about what happened and to face hard questions about existential issues or about death. Being accessible was important not only in the acute phase but also in the longer term by offering follow-up visits or contact and being there for anniversaries. However, it was always up to the child to decide whether the school nurse was the adult to confide in.

Being humble and respecting the wishes of the child and their family was important for the school nurse. Being there for the child meant really listening and being responsive to when the child wanted to talk and what they wanted to talk about. It required attention to the individual needs of the child and was described as a delicate balance between what was unspoken and what was spoken. Extra attention from the school nurse was required as some children came with other symptoms when in fact they needed emotional support:

Meeting the student when he is consulting about other things but probably really wants some human contact and maybe a smile. Seeing and listening to what is also behind the surface is important in my role. (Participant no. 40, female, two years of experience)

Being attentive to the needs of the child was fundamental to helping the child make the grief manageable. Normalising, confirming, enabling, and putting feelings into words were among the strategies used by the school nurse. On one hand, it could be about supporting the child in allowing themselves to feel happy. On the other hand, it could be about normalising and putting words to feelings of guilt about losing a family member:

Let the child know that I am here, that they are not alone, that grief can be felt in different ways, and that everything is ok. Some children are ashamed because, for example, a parent has died; if so, it is important to normalise their feelings. (Participant no. 13, female, four years of professional experience)

Having a previous relationship with the child through regular health talks improved the possibilities for the school nurse to support the child effectively. Regular health talks/health examinations were a unique opportunity to build relationships with every child. School nurses stressed the importance of continuing to promote the health of the child, in terms of basic needs like sleep, eating, and physical activity. Promoting health could also be about helping to identify things that could bring joy and meaning for the child, aiding them in enduring and coping with the grief:

. . .Encourage the healthy, encourage play, gauge what the child thinks is fun. In order to be able to grieve, the child also needs to experience joy and meaning in his everyday life. (Participant no. 57, female, one year of professional experience)

## Discussion

The vast majority of school nurses in this study reported that they felt secure in their professional role when meeting children in grief. They had acquired preparedness through previous private and professional experiences of grief and through nursing and specialist education. This is in line with previous research reporting that training for school nurses to support grieving children was significantly and positively correlated with a higher level of school support for the grieving child. In the same study, school nurses with higher education were less likely to report personal distress when supporting grieving children (Demaria et al., 2023). On the other side, previous research has also highlighted that poor preparation in terms of lacking formal training leaves school nurses with inadequate tools to meet and care for children in grief (Auman, 2007; Edwards et al., 2023; Lohan, 2006). Taken together, the importance of the school nurse’s professional experiences can be linked to Barbara Dossey’s holistic nursing theory with the core focus on healing. The theory is suggested to be useful within several areas of school nursing (Garmy et al., 2021). The theory focuses on not only the patient/student but also on the nurse as being a part of the healing process. One part in the holistic nursing theory is the nurse’s different patterns of knowing, with different areas of knowledge in school nursing in this case. Professional experiences are a part of empirical knowledge, whereas the focus on exploring experiences and meaning in life is a part of aesthetic knowledge. These are areas of knowledge that can help the school nurse to be present in the present and thereby being a facilitator in the healing process (Garmy et al., 2021). Thus, educational preparation, training, and previous experiences of meeting grieving children (and adults) are probably key factors in preparing school nurses in their role to provide support for grieving children in school settings.

Access to regular professional guidance is essential to prevent emotional overload.

The importance of being there and continuing the work to promote the health of the grieving child was stressed by the participants in our study. The school nurses’ health-promoting work can help the child through identifying and meeting their social, existential, and physical needs. Healthy mourning is based on not only maintaining and nurturing healthy daily rituals but also giving children permission to grieve (Riely, 2003). Being there for the child as a school nurse applies through all the phases of grieving, helping the child in the dynamic and iterative process on the path to acceptance and adaptation (Kübler-Ross, 1997). The participants stressed that to continue health-promoting work during the grief was built on having a prior relationship and knowing the child. Knowing the child was especially needed in situations when the child came to the school nurse with physical symptoms, but it turned out they needed emotional support. An existing relationship with the student enables the school nurse to discover the real reason for the visit. Demaria et al. (2023) described it as when grief shows up in the school nurse’s office as ‘tummy aches and headaches’.

Being there for the child was also about being humble, respecting the wishes of the child, and being flexible regarding the type of support and how and when to provide it. Support could be about normalising, confirming, enabling, putting feelings into words, or having the courage to face existential questions. Responsiveness to the child was expressed as vital. The importance of adjusting both attention and communication with bereaved children is supported by previous studies (Demaria et al., 2023; Lytje 2018; Morell-Velasco et al. 2020), and further highlighted by research showing that poor communication is a modifiable risk factor for adverse psychosocial consequences among bereaved children (Hoffman et al., 2018). School is a central part of the grieving child’s everyday life both before and after their loss, having the potential to be a valuable resource of support in this respect (Hoffman et al., 2018, Lytje, 2018). Continuing to be a central part of everyday life can support the child’s need for normality, their need to be with peers, and to not stand out from the crowd (Erikson, 1977). However, for the return to school to be successful including the child and being responsive to their wishes seems to be of the utmost importance (Lytje 2018). Support for grieving children must include collaboration with teachers and mental health professionals. In accordance with the guidance document for student health, students and guardians can contact, for example, the school nurse or counsellor if they have questions about mental health (National Board of Health and Welfare, 2023). The school nurse, psychologist, and counsellor can have various types of supportive conversations with individual students for a limited period. However, confidential rules restrict the contact between various healthcare providers and school nurses which means that there are no obvious pathways for information transfer to the child’s school nurse. In a study by Golsäter et al. (2017), school nurses describe that often parents and teachers believe the school nurse works in conjunction with other health providers. Increased cooperation is needed between the school nurses and teachers to be able to detect children in grief and thus be able to provide optimal support. Routines and collaboration to recognise the child when his/her parent has become ill is described as crucial to accomplishing this assignment. A clear definition of roles and responsibilities as well as the opportunities to collaborate with other health professionals and other members of the school health team is important.

### Strengths and Limitations

Using a web-based survey for data collection enabled wide distribution of the survey and there was good representation across geographical areas and experience. However there were some methodological limitations. One was that it was not possible to analyse the external attrition. We do not have any data about how many potential participants chose not to participate, and why they did not participate or any data about them. Knowledge about potential selection bias is also limited, as we do not know anything about the school nurses who were not invited to participate (not on social media, not attending the conference). Although a small group of participants replied that they had no experience of meeting children in grief, we choose to still analyse their answers as they reflected thoughts related to feeling prepared or not. Furthermore, open questions were used in the survey, but there was no opportunity for follow-up questions as are often used in qualitative interviews. Given the limitations addressed above, the transferability of the results consequently is limited to school nurses and contexts represented by the participants in this study. However, considering the total number of responses and the amount of text that was analysed we believe that the advantages of the web-based survey to some extent outweighed the limitations in this study.

## Conclusion

The results from this study show that the school nurse can play an important role for children in grief due to the loss of a family member. Meeting with the grieving child requires presence and responsiveness from the school nurse. Based on their professional knowledge and experience of encountering children, the school nurse’s supportive role can include normalising and confirming the child’s position, thereby helping the school reflect a secure environment for the child. The vital role that school nurses play in supporting grieving children can be both recognised and strengthened by the rest of the student healthcare team, teachers, and school management. By equipping school nurses with the resources they need to establish relationship with the children, along with preparational training, it can be ensured that children in grief feel seen, supported, and secure within the school environment. Further research should focus on school nurses’ more specific needs for preparational training and on their need for support during their work with children in grief.

**Key points for policy, practice and/or research**

- The role of the school nurse can be an accessible specialist practitioner for the bereaved child in school. This includes listening and being responsive when the child wants to talk, and what they want to talk about.
- Specialist knowledge about grief and grief among children, and previous clinical experience create security for the school nurse in the meeting with the bereaved child, as well as being part of a supoortive team.
- Having a previously established trusted relationship with the child through regular health talks improves the possibilities for the school nurse to support the child.
- School nurses can play a central role in creating a secure environment for the bereaved child, with as much normality as possible in keeping with child development.
